# Edge-guided second-order total generalized variation for Gaussian noise removal from depth map

**DOI:** 10.1038/s41598-020-73342-3

**Published:** 2020-10-01

**Authors:** Shuaihao Li, Bin Zhang, Xinfeng Yang, Weiping Zhu

**Affiliations:** 1 Research Center for International Business and Economy, Sichuan International Studies University, Chongqing, 400031 China; 2 International Business School, Sichuan International Studies University, Chongqing, 400031 China; 3 School of Computer Science, Wuhan University, Wuhan, 430072 China; 4 Department of Computer Science, City University of Hong Kong, Hong Kong, 999077 China

**Keywords:** Imaging and sensing, Optical sensors

## Abstract

Total generalized variation models have recently demonstrated high-quality denoising capacity for single image. In this paper, we present an accurate denoising method for depth map. Our method uses a weighted second-order total generalized variational model for Gaussian noise removal. By fusing an edge indicator function into the regularization term of the second-order total generalized variational model to guide the diffusion of gradients, our method aims to use the first or second derivative to enhance the intensity of the diffusion tensor. We use the first-order primal–dual algorithm to minimize the proposed energy function and achieve high-quality denoising and edge preserving result for depth maps with high -intensity noise. Extensive quantitative and qualitative evaluations in comparison to bench-mark datasets show that the proposed method provides significant higher accuracy and visual improvements than many state-of-the-art denoising algorithms.

## Introduction

Since the advent of ToF sensor, the denoising research on the depth map acquired by it has never stopped. As a major noise type in depth map, Gaussian noise has been widely concerned by researchers. Consequently effective denoising methods have appeared, such as, filtering-based methods^1–3^, partial differential equation (PDE)-based methods^4–6^, sparse representation-based dictionary learning methods^7–11^, deep learning-based methods^12–20^, and recent variation minimization-based methods^21–24^.

The filtering methods mainly include spatial domain filtering and frequency domain filtering. No matter which filter, there are some common phenomena such as incomplete denoising, blur edge, and inconsistency between the smooth area of image denoised and the constant area of ground truth.

The PDE-based methods can diffuse the smooth area of image quickly and the edge area slowly by setting diffusion factors, and finally achieve the effect of denoising and edge preservation. However, for images polluted by high-density noise, the denoising effect of the PDE-based methods is poorly achieved as the termination of the iterative process is hard to control and the convergence speed is slow causing the poor real-time performance.

For sparse representation-based methods, their performance mainly depends on image sparse domain. But, in practice, it is usually not easy to choose the sparse domain due to the variety of images.

The deep learning-based methods have recently demonstrated high-quality denoising for various images. However, such methods also have some drawbacks, such as, the optimization of network structure requires a lot of training, and the datasets required for training are often very large. In addition, specific models need to be trained for specific noise intensity, so their application universality is still poor.

The variation minimization-based methods denoise by iterating the variational model composed of regularization term and data item. The most representative one of this type of method is the total generalized variation (TGV) model proposed by Bredies et al.^24^. The TGV model can not only remove noise effectively, but also preserve the detailed feature of image edge well. However, when the image edge is polluted by noise heavily, the TGV model is prone to misjudge the noises as the edge and therefore unable to extract the noises from the edge.

The goal of our work is to denoise depth maps polluted by high-intensity Gaussian noise and effectively remove the noises to preserve the edges. In this work, we have presented an edge-guided second-order total generalized variation for Gaussian noise removal from depth map (ESTGV). In the ESTGV model, the edge indicating function Canny algorithm is added into the regularization term of the second-order generalized total variational, which can guide the diffusion of the gradients, and then adaptively select the first or second derivative to update the diffusion tensor strength according to the edge indicator function value in the edge area and the smooth area. Then the minimum approximation of the energy function is solved by the first-order primal–dual algorithm (FOPD)^25^, which can effectively preserve the edge by denoising the depth map with high-density Gaussian noise. The experimental results showed that the ESTGV outperforms state-of-the-art denoising methods not only in PSNR index, but also in visual comparison.

## Related work

### Total variation (TV) for image denoising

The total variation model (TV) is a classical image denoising algorithm proposed by Rudin et al.^22^, also known as the ROF model. The minimum energy functional of the TV model can be expressed as:

1 $$\mathop {\min }\limits_{\Omega } a\int\limits_{\Omega } {|\nabla u|} dxdy + \frac{\lambda }{2}\int\limits_{\Omega } {(u - f)^{2} } dxdy,$$

where $u$ denotes the predicted image, $u \in \Omega \in R$, $f$ denotes the noisy image, and the regularization term $\int\limits_{\Omega } {|\nabla u|} dxdy$ denotes the prior constraint of image $u$, which is used for image denoising. $|\nabla u|$ denotes the model of the gradient of $u$. $\frac{\lambda }{2}\int\limits_{\Omega } {(u - f)^{2} } dxdy$ is the data fidelity term, also known as the approximation term, which measures the approximation degree of $u$ and $f$ by distance and constrains the solution of total variation minimizing to limit the deviation of solution in a small range, so as to achieve the purpose of protecting the structural information of image and reducing the distortion of image. $\alpha$ and $\lambda$ are Lagrange scale constants for adjusting regularization term and data fidelity term. They need a proper balancing between the edge preservation and the noise removal. The larger $\lambda$ is, the closer $f$ approaches $u$, but if the value of $\lambda$ is too large, the smoothing intensity of local features will be weakened, and the denoising effect will be decreased; the smaller $\lambda$ is, the greater the denoising degree will be, but if the value of $\lambda$ is too small, the image will become blurred.

The Euler–Lagrange equation of energy functional (1) can be expressed as:

2 $$- \nabla \cdot \left( {\frac{{\nabla u}}{{\left| {\nabla u} \right|}}} \right) + \lambda (u - f) = 0,$$

where the first term $- \nabla \cdot \left( {\frac{{\nabla u}}{{\left| {\nabla u} \right|}}} \right)$ is the diffusion tensor and $\frac{1}{{\left| {\nabla u} \right|}}$ is the diffusion coefficient. In the edge area with large gradient, $\left| {\nabla u} \right|$ is larger, the diffusion coefficient is small, and the diffusion intensity along the edge direction is smaller, so the edge detail features can be well preserved. In the smooth area with small gradient, $\left| {\nabla u} \right|$ is smaller, the diffusion coefficient is higher, the denoising intensity is higher.

The TV model determines the edge and the noise by the gradient value, and the diffusion is performed only in the gradient orthogonal direction of the image, so that the edge structure can be effectively preserved. But in the smooth area of the image, the TV model can only approximate the piecewise constant function effectively, so it is easy to misjudge the noise as edge to be preserved, and eventually produce the “staircasing artifacts” of the piecewise area.

### Second-order total generalized variation

In order to solve the problem of “staircasing artifacts” in denoising of TV model, Bredies et al.^24^ proposed a generalized mathematical model of TV algorithm, that is, total generalized variation (TGV). Different from the fact that the TV model can only approximate the piecewise constant function, the convex optimization model generated by the TGV model can approximate the piecewise multinomial function of any order, thus effectively avoiding the “staircasing artifacts” in the process of denoising.

The k-order TGV model can be defined as:

3 $$TGV_{\alpha }^{k} (u) = \sup \left\{ {\int_{\Omega } {u{\text{ div}}^{k} v \, dx \, \left| { \, v \in c_{c}^{k} \left( {\Omega ,Sym^{k} \left( {{\mathbb{R}}^{d} } \right)} \right), \, \left\| {{\text{div}}^{l} v} \right\|_{\infty } \le \alpha_{l} ,l = 0, \cdots ,k - 1} \right.} } \right\}$$

where $$\Omega$$ is an open interval, $$\Omega \in {\mathbb{R}}^{d}$$, and $$u$$ denotes the original image, $$u \in L_{loc}^{1} \left( \Omega \right)$$,$$TGV_{\alpha }^{k}$$ denotes the k-order total generalized bounded variations with the weight coefficient $$\alpha$$($$k \ge 1$$$$\alpha = \left( {\alpha _{0} , \cdots ,\alpha _{{k - 1}} } \right)0$$), and $$TGV_{\alpha }^{k}$$ provides a way of automatically balancing between the first derivative and the *k*-order derivative. $${\text{div}}^{k} v$$ denotes the *k*-order symmetric divergence operator. $$v \in c_{c}^{k} \left( {\Omega ,Sym^{k} \left( {{\mathbb{R}}^{d} } \right)} \right)$$ denotes the functions of *k*-order symmetric tensor in the $$\Omega$$, where $$c_{c}^{k}$$ is *k*-order continuous function space, $$Sym^{k} \left( {{\mathbb{R}}^{d} } \right)$$ is *k*-order symmetric tensor space based on $${\mathbb{R}}^{d}$$, and $$\alpha_{l}$$ is a fixed positive parameter.

When $k = 1$ and $\alpha_{1} = 1$, Eq. (3) is the dual form of TV model:

4 $$TGV_{1}^{1} (u) = \sup \left\{ {\int_{\Omega } {u{\text{ div }}v{\mkern 1mu} dx{\mkern 1mu} \left| {\left\| v \right\|_{\infty } \le 1} \right.} } \right\} = TV\left( u \right)$$

According to Legendre–Fenchel convex conjugate transformation^26^, the original form of formula (3) can be obtained:

5 $$TGV_{\alpha }^{k} (u) = \mathop {\inf }\limits_{\begin{subarray}{l} u_{l} \in C_{c}^{k - l} \left( {\Omega ,Sym^{l} \left( {{\mathbb{R}}^{d} } \right)} \right) \\ l = 1, \cdots ,k - 1,u_{0} = u,u_{k} = 0 \end{subarray} } \sum\limits_{l = 1}^{k} {\alpha_{k - 1} \left\| {\varepsilon (u_{l} - 1) - u_{l} } \right\|_{1} } ,$$

where the gradient operator $\varepsilon (u_{l} - 1)$ denotes symmetric partial derivative, which can be expressed as:

6 $$\varepsilon (u_{l} - 1) = \frac{1}{2}\left( {\nabla u_{l - 1} + \left( {\nabla u_{l - 1} } \right)^{T} } \right).$$

In particular, for $k = 2$, Eq. (3) is the second-order TGV:

7 $$TGV_{\alpha }^{2} (u) = \mathop {\min }\limits_{\omega \in BD\left( \Omega \right)} \alpha_{1} \int_{\Omega } {\left| {\nabla u - \omega } \right|} \, dx{ + }\alpha_{0} \int_{\Omega } {\left| {\varepsilon \left( \omega \right)} \right.} \left| {dx} \right.$$

where $BD\left( \Omega \right)$ is a bounded and distorted vector field^27^, the gradient operator $\varepsilon (\omega ) = \frac{1}{2}\left( {\nabla u_{\omega } + \nabla u^{T} } \right)$_._

The corresponding second-order denoising model is expressed as:

8 $$\mathop {\min }\limits_{u} TGV_{\alpha }^{2} \left( u \right){ + }\frac{{\left\| {u - f} \right\|_{2}^{2} }}{2}$$

The TGV model can effectively avoid the “staircasing artifacts” of piecewise constant areas and is superior to TV model in denoising and edge preservation.

### The first-order primal–dual algorithm (FOPD)

The FOPD algorithm is a particularly effective iterative algorithm proposed by Chambolle et al.^25^ in 2011, which is used to solve the convex optimization problems with a special proximal regularization parameter. The FOPD algorithm converts the original minimizing problem into the dual maximization problem by dual variables, and then represents the original problem and the dual problem as their corresponding saddle-point optimization problem, and then iterates the original variables and dual variables alternately until the optimal solution of the original problem is finally approximated. The FOPD algorithm is easy to implement and can effectively iterate alternately, so it can accelerate convergence.

The general convex optimization problem is defined as:

9 $${\mathop {\min }\limits_{x \in X} F\left( {Ix} \right) + \text{ G}}\left( x \right),$$

the corresponding dual problem can be expressed as:

10 $$\mathop {\max }\limits_{y \in Y} - \left\{ {F^{ * } \left( y \right){\text{ } + \text{ G}}^{ * } \left( { - I^{ * } y} \right)} \right\},$$

where $x$ is the primal variable, $y$ is the dual variable. $X$ and $Y$ denote the finite-dimensional real vector space of the image with an inner product $\langle \cdot , \cdot \rangle$ and norm $\left\| \cdot \right\| = \sqrt {\langle \cdot , \cdot \rangle }$, $I:X \to Y$ is a continuous linear operator, $F\left( {Ix} \right)$ and ${\text{G}}\left( x \right)$ are semi-continuous convex functions, $F^{ * }$ and $G^{ * }$ are conjugate convex functions that are topologically dual to functions $F$ and $G$, respectively. $I^{ * }$ is the adjoint operator of $I$.

The saddle-point formulation of (9) and (10) is given by

11 $$\mathop {\min }\limits_{x \in X} \mathop {\max }\limits_{y \in Y} \left[ {Ix,y} \right] + G\left( x \right) - F^{ * } \left( y \right).$$

For $F\left( {If} \right) = \left\| {\nabla f} \right\|_{1}$ and ${\text{G}}\left( f \right) = \frac{\lambda }{2}\left\| {f - g} \right\|_{2}^{2}$, (11) becomes

12 $$\mathop {\min }\limits_{f \in X} \mathop {\max }\limits_{y \in Y} \left\{ {\langle \nabla f,y\rangle_{Y} + \frac{\lambda }{2}\left\| {f - g} \right\|_{2}^{2} - \delta_{P} \left( y \right)} \right\},$$

where, the function $\delta_{P} \left( y \right)$ is the dual function of $F\left( g \right) = \left\| g \right\|_{1}$:

13 $$F^{*} \left( y \right) = \delta _{P} \left( y \right) = \left\{ {\begin{array}{*{20}l} 0 & {{\mkern 1mu} \left\| y \right\|_{\infty } \le 1} \\ \infty & {otherwise} \\ \end{array} } \right.$$

The convex set *P* is expressed as:

14 $$P = \left\{ {p \in Y:\left\| p \right\|_{\infty } \le 1} \right\}$$

$\left\| p \right\|_{\infty }$ denotes the discrete maximum norm defined as:

15 $$\left\| p \right\|_{\infty } = \max \left| {p_{i,j} } \right|,\left| {p_{i,j} } \right| = \sqrt {\left( {p_{i,j}^{1} } \right)^{2} + \left( {p_{i,j}^{2} } \right)^{2} }$$

According to the projection approximation algorithm, the iteration formulas of the original variable $x$ and the dual variable $y$ can be expressed as:

16 $$\begin{gathered} y^{n + 1} = (I + \sigma \partial F)^{ - 1} (y^{n} + \nabla \overline{f}^{n} ) \hfill \\ \Leftrightarrow \, y^{n + 1} = \arg \, \mathop {\min}\limits_{y \in Y} \left\{ {\frac{1}{2\sigma } \cdot \left\| {y - (y^{n} + \sigma \nabla \overline{f}^{n} )} \right\|_{2}^{2} + \delta (y)} \right\} \hfill \\ \Leftrightarrow \, y_{i,j}^{n + 1} = \left( {y_{i,j}^{n} + \sigma \nabla \overline{f}_{{^{i,j} }}^{n} } \right) \cdot \max \left( {1,\left| {y_{i,j}^{n} + \sigma \nabla \overline{f}_{{^{i,j} }}^{n} } \right|} \right)^{ - 1} , \hfill \\ \end{gathered}$$

and

17 $$\begin{gathered} f^{n + 1} = (I + \tau \partial G)^{ - 1} (f^{n} + \tau {\text{div }}y^{n + 1} ) \hfill \\ \Leftrightarrow \, f^{n + 1} = \arg \, \mathop {\min}\limits_{f} \left\{ {\frac{1}{2\tau } \cdot \left\| {f - (f^{n} + \tau {\text{div }}y^{n + 1} )} \right\|_{2}^{2} + \frac{\lambda }{2}\left\| {f - g} \right\|_{2}^{2} } \right\} \hfill \\ \Leftrightarrow \, f_{i,j}^{n + 1} = \left( {\left( {f_{i,j}^{n} + \tau {\text{div }}y_{i,j}^{{^{n + 1} }} } \right) + \tau \lambda g_{i,j} } \right) \cdot \left( {1 + \tau \lambda } \right)^{ - 1} , \hfill \\ \end{gathered}$$

and

18 $$\overline{f}^{n + 1} = f^{n + 1} + \theta \left( {f^{n + 1} - f^{n} } \right) = 2f^{n + 1} - f^{n} .$$

## The proposed method

### Edge-guided second-order total generalized variation for Gaussian noise removal

In order to solve the edge blurring issue during denoising depth maps with high-intensity Gaussian noise, we propose an edge-guided second-order total generalized variation model (ESTGV) for depth map Gaussian noise removal. The ESTGV model aims to preserve the depth edge and detail features profoundly when denoising Gaussian noise heavily polluted depth maps by adding an edge indication function into the second-order total generalized variation model.

For the choice of edge indication function, we have compared some other edge detection algorithms, and found that Roberts detector usually misses some edges, Sobel detector and Prewitt detector often generate false edges, Laplacian detector is over-sensitive to noise and not suitable for noisy images, and LOG (Laplacian of Gaussian) detector usually loses sharp edges, while Canny detector adopts the principle of optimal edge detection, which has strong anti-noise ability, high integrity and continuity of edge detected , and adaptive threshold generation, and its edge preserving performance is obviously better than other detectors.

By adding the Canny edge detection algorithm^28^ into the regularization term of the second-order TGV, we propose a weighted second-order TGV minimization denoising model:

19 $$\mathop {\min }\limits_{\Omega } (u) + \frac{\lambda }{2}\int\limits_{\Omega } {(u - f)^{2} } dx$$

where $\mathop {\min }\limits_{\Omega } (u)$ is the regularization term, $\frac{\lambda }{2}\int\limits_{\Omega } {(u - f)^{2} } dx$ is the data fidelity term, $u$ denotes the predicted depth map denoised, $f$ denotes the noisy depth map, $\lambda$ is the Lagrange constant factor for balancing the regularization term and the data fidelity term, $\Omega$ is the definition domain of pixels on the depth map, $(x,y) \in \Omega$.

The regularization term $\mathop {\min }\limits_{\Omega } (u)$ can be expressed as $\Phi (u)$, that is, the second order total generalized variation $TGV_{a}^{2} (u)$:

20 $$\Phi (u) = TGV_{a}^{2} (u) = \mathop {\min }\limits_{p} a_{1} \parallel \varepsilon (p)\parallel _{1} + a_{2} \parallel T(\nabla u - p)\parallel _{1}$$

where $p \in R^{mn} \times R^{mn}$ is a bounded two-dimensional first-order vector field, $p_{(i,j)} = [p_{i,j,1} ,p_{i,j,2} ]$. $a_{1}$ and $a_{2}$ are two constants used to coordinate the first derivative and second derivative; $\nabla u$ is the gradient map of $u$, which is used to judge the scale of noise and edge. The gradient formulation of pixel $u\left( {x,y} \right)$ on depth map $u$ is given by:

21 $$\left| {\nabla u(x,y)} \right|_{n} = \sqrt {\left( {\frac{{\partial u}}{{\partial x}}} \right)_{n}^{2} + \left( {\frac{{\partial u}}{{\partial y}}} \right)_{n}^{2} }$$

where $\left( {\frac{\partial u}{{\partial x}}} \right)_{n}$ and $\left( {\frac{\partial u}{{\partial y}}} \right)_{n}$ denote the horizontal and vertical gradients in the *nth* iteration process, respectively, which can be calculated by the median difference:

22 $$\left\{ \begin{gathered} \left( {\frac{\partial u}{{\partial x}}} \right)_{n} = \frac{1}{2}\left( {u\left( {x + 1,y} \right)_{n} - u\left( {x - 1,y} \right)_{n} } \right) \hfill \\ \left( {\frac{\partial u}{{\partial y}}} \right)_{n} = \frac{1}{2}\left( {u\left( {x,y + 1} \right)_{n} - u\left( {x,y - 1} \right)_{n} } \right) \hfill \\ \end{gathered} \right.$$

$T$ of (20) denotes the edge indicator function:

23 $$T = \frac{1}{{1 + M|\nabla G_{\sigma } * f|^{2} }}$$

where M ≥ 0 is the contrast factor, $G_{\sigma }$ is the Gaussian filtering kernel with a mean value of 0 and a standard deviation of σ. *denotes convolution operation, and $G_{\sigma } *f$ denotes the depth map preprocessed by Gaussian filtering kernel $G_{\sigma } *f$.

$\varepsilon (p)$ in (20) denotes the second-order symmetric gradient operator:

24 $$\varepsilon (p) = \frac{{\nabla p + (\nabla p)^{T} }}{2} = \left[ \begin{gathered} \varepsilon (p)_{i,j,1 \, } \, \varepsilon (p)_{i,j,3} \hfill \\ \varepsilon (p)_{i,j,3} \, \varepsilon (p)_{i,j,2} \hfill \\ \end{gathered} \right]$$

The regularization item $G_{\sigma } (x)$ is the Canny edge detection algorithm:

25 $$G_{\sigma } (x) = \frac{1}{{2\pi \sigma^{2} }}\exp [ - \frac{{x^{2} + y^{2} }}{{2\sigma^{2} }}]$$

where σ is the weight of the Gaussian filter to adjust the smoothing level. The smaller the σ value, the higher the positioning accuracy of the filter, but the lower the signal-to-noise ratio (SNR). The larger the σ value, the higher the SNR, but the positioning accuracy of the filter is also reduced.

According to the denoising model (19), the edge detection of the depth map is performed by $|\nabla G_{\sigma } *f|^{2}$, if the value of $|\nabla G_{\sigma } *f|^{2}$ is large, the position of the observed pixel is the depth edge, and the value of T decreases because of the larger denominator, so the first derivative diffusion becomes relatively weak, so that the edge structure can be preserved well. If the value of $|\nabla G_{\sigma } *f|^{2}$ is small, the observed pixel is located in the smooth area of the depth map, the value of $T$ is close to 1 and the diffusion degree of the first derivative increases, so that the noise can be filtered effectively.

In conclusion, the edge indicator function can adaptively adjust the diffusion intensity of different frequency domains in depth images. For the edge areas, the first derivative is used to preserve their detailed features. For the smooth areas, the higher order derivative is used to filter out the noise, so as to effectively preserve the edge while denoising the depth map with high-density gaussian noise.

### Numerical algorithms

In our denoising model, the regularization term is proper, convex and lower semi-continuous, and the data fidelity term is strictly convex and completely non-smooth. According to the convex analysis theory^29^, (19) is an unconstrained optimization problem, and there is a unique minimum solution, that is, the global optimal solution^30^.

We employ the FOPD method^25^ as the numerical algorithm in order to solve the ESTGV model (19). The FOPD algorithm can solve optimization problems with efficient iterations.

According to the Legendre-Fenchel transformation^24^, the dual form of (19) can be obtained from:

26 $$\mathop {\min }\limits_{u,w} \mathop {\max }\limits_{m \in M,n \in N} \langle \nabla u - p,m\rangle + \langle \varepsilon (p),m\rangle + \frac{\lambda }{2}\left\| {(u - f)^{2} } \right\|_{2}^{2}$$

where

27 $$\begin{gathered} M = \{ m = (m_{1} ,m_{2} )^{T} ||m(x)| \le Ta_{2} \} , \hfill \\ N = \left\{ {n = \left( \begin{gathered} n_{11} {, }n_{12} \hfill \\ n_{21} , \, n_{22} \hfill \\ \end{gathered} \right)\left| {\left\| n \right\|_{\infty } \le a_{1} } \right|} \right\} \hfill \\ \end{gathered}$$

Using the original-dual algorithm^25^ to solve (26), The iterative process is obtained:

28 $$\left\{ \begin{gathered} m^{k + 1} = proj_{M} (m^{k} + a(\nabla \overline{u}^{k} - \overline{p}^{k} )) \hfill \\ n^{k + 1} = proj_{N} (n^{k} + a(\varepsilon (\overline{p}^{k} ))) \hfill \\ u^{k + 1} = (u^{k} + \tau ({\text{div }}m^{k + 1} + \lambda f)) \cdot (1 + \tau \lambda ) \hfill \\ p^{k + 1} = p^{k} + \tau ({\text{div}}^{h} \, n^{k + 1} + m^{k} ) \hfill \\ \overline{u}^{k + 1} = u^{k} + \eta (u^{k + 1} - \overline{u}^{k} ) \hfill \\ \overline{p}^{k + 1} = p^{k + 1} + \eta (p^{k + 1} - \overline{p}^{k} ) \hfill \\ \end{gathered} \right.$$

where $k$ denotes the number of iterations, the divergence operator $div$ is the dual operator of the gradient operator $\nabla$, which denotes the negative conjugate of the gradient operator $\nabla$, i.e. $div = - \nabla*$, the discrete version of $div$ is $div(m_{1} ,m_{2} ) = \partial_{x}^{ - } m_{1} + \partial_{y}^{ - } m_{2}$,$div^{ h}$ denotes the negative conjugate of the second-order symmetric gradient operator $\varepsilon$, $div^{h} = - \varepsilon*$. When

$$m = \left( {\begin{array}{*{20}l} {m_{{11}} ,} & {m_{{12}} } \\ {m_{{21}} ,} & {m_{{22}} } \\ \end{array} } \right),\;div^{h} (n) = \left( {\begin{array}{*{20}c} {\partial _{x}^{ - } m_{{11}} + \partial _{y}^{ - } m_{{12}} } \\ {\partial _{x}^{ - } m_{{21}} + \partial _{y}^{ - } m_{{22}} } \\ \end{array} } \right).$$

$\partial_{x}^{ - } ,\partial_{y}^{ - }$ is the first-order backward differences operator.

The projections of (24) can be easily obtained by applying pointwise operations below:

29 $$\left\{ {\begin{array}{*{20}l} {proj_{M} (m){\text{ }} = {\text{ m}} \cdot (max\left( {1,\frac{{\left| m \right|}}{{Ta_{2} }}} \right)^{1} } \\ {proj_{N} (n) = {\text{ n}} \cdot (max\left( {1,\frac{{\left| n \right|}}{{Ta_{1} }}} \right)^{1} } \\ \end{array} } \right.$$

In conclusion, the solution of denoising model (19) is an iterative process for solving original variables $u,p$ and dual variables $m,n$. The specific solving process of the primal–dual algorithm is as follows:

*Step 1:* Initialization:

$$\begin{aligned} & u^{0} = \bar{u}^{0} = f,p^{0} = \bar{p}^{0} = 0,m^{0} = n^{0} = 0, \\ & \eta _{m} > 0,\eta _{n} > 0,\tau _{u} > 0,\tau _{p} > 0,\lambda > 0,k \ge 0. \\ \end{aligned}$$

*Step 2:* Using the Iteration formulation (22) to solve $m^{j,k + 1} ,n^{j,k + 1} ,u^{j,k + 1} ,p^{j,k + 1} ,\overline{u}^{j,k + 1} ,\overline{p}^{j,k + 1}$.

*Step 3:* When $u^{k + 1} ,p^{k + 1}$ appear to converge, the algorithm is terminated; otherwise, set $k = k + 1$, and continue to iterate until the stopping criterion is satisfied.

The numerical operation of the ESTGV model (19) needs to be implemented in a discrete version of the gradient operator and the divergence operator.

Assuming that the resolution of depth map is s × s, then the discrete versions of the first-order forward and backward difference operators are given by

30 $$\begin{aligned} (\partial _{x}^{ + } u)_{{i,j}} & = \left\{ {\begin{array}{*{20}l} {u_{{i + 1,j}} - u_{{i,j}} ,} \hfill & {1 \le i < n} \hfill \\ {0,} \hfill & {i = n} \hfill \\ \end{array} } \right. \\ (\partial _{y}^{ + } u)_{{i,j}} & = \left\{ {\begin{array}{*{20}l} {u_{{i,j + 1}} - u_{{i,j}} ,} \hfill & {1 \le i < n} \hfill \\ {0,} \hfill & {j = n} \hfill \\ \end{array} } \right. \\ (\partial _{x}^{ + } u)_{{i,j}} & = \left\{ {\begin{array}{*{20}l} {u_{{i,j}} - u_{{i - 1,j}} ,} \hfill & {1 < i < n} \hfill \\ {u_{{1,j}} } \hfill & {i = 1} \hfill \\ { - u_{{n - 1,j}} ,} \hfill & {i = n} \hfill \\ \end{array} } \right. \\ (\partial _{y}^{ + } u)_{{i,j}} & = \left\{ {\begin{array}{*{20}l} {u_{{i,j}} - u_{{i,j - 1}} ,} \hfill & {1 < j < n} \hfill \\ {u_{{j,1}} ,} \hfill & {j = 1} \hfill \\ { - u_{{i,n - 1}} ,} \hfill & {j = n} \hfill \\ \end{array} } \right. \\ \end{aligned}$$

The discrete version of the gradient operator is expressed as $\nabla = \left( {\partial _{x}^{ + } ,\partial _{y}^{ + } } \right)^{T}$, and the discrete second-order symmetric gradient operator $\varepsilon$ is expressed as:

31 $$\varepsilon (p) = \frac{1}{2}(\nabla p + \nabla p^{T} ) = \left[ \begin{gathered} \partial_{x}^{ + } p_{1} ,\frac{1}{2}(\partial_{y}^{ + } p_{1} + \partial_{x}^{ + } p_{2} ) \hfill \\ \frac{1}{2}(\partial_{x}^{ + } p_{2} + \partial_{y}^{ + } p_{1} ),\partial_{y}^{ + } p_{2} \hfill \\ \end{gathered} \right]$$

## Experimental results

In this section, we evaluate our proposed method both quantitatively and qualitatively with respect to other benchmark and state-of-the-art image denoising methods.

***Experimental platform:*** The simulations are carried out on MATLAB R2018a using a laptop with a quad-core 2.2 GHz Intel(R) i7-4770HQ CPU, 16 GB RAM, Intel (R) Iris (TM) Pro Graphics 5200.

***Experimental Dataset:*** We experimented with our denoising method on the public dataset, namely the Middlebury stereo dataset^31,32^. The resolution of each depth map is 256 × 256.

***Baseline Methods:*** We compare our results with the following *five* categories of the methods. (1) *Filtering-based methods*: Bilateral Filter (BF)^1^ and Block-Matching and 3D Filtering (BM3D)^2^. (2) *PDE-based methods:* Weighted Nuclear Norm Minimization (WNNM)^6^. (3) *Sparse representation algorithm*: K-Singular Value Decomposition (K-SVD)^7^. (4) *Deep learning-based methods* including Multi-Layer Perceptron (MLP)^12^, Trainable Nonlinear Reaction Diffusion (TNRD)^13^ and A Cascade of Shrinkage Fields (CSF)^14^. (5) *Variational-based methods:* Total Variation (TV)^22^ and Total Generalized Variation (TGV)^24^. The results of the comparison algorithms are either obtained from the original papers or from the source code provided by the authors.

***Parameters Setting:*** The parameters of our ESTGV algorithm are set as follows: Lagrange multiplier $\lambda = 10$,Gaussian noise of standard deviation $\sigma = 0.2$, contrast factor *M* = 5, filter kernel size $7 \times 7$, $a_{2} = 2$, $a_{1} = 4$,$\tau = 0.04$. We also set parameters for each comparison method. Note that at least one of these parameters is from the default parameters provided by the authors, while the others are selected by ourselves.

### Quantitative results

We first evaluate our results on six benchmark maps (Art, Books, Dolls, Laundry, Moebius and Reindeer) from Middlebury Stereo dataset^32^. Note that we added Zero-Mean-Gaussian noise with standard deviation 15, 20, 25 and 50 to all depth maps. We use peak signal-to-noise ratio (PSNR) as the evaluation metric. Tables 1 and 2 show the comparison of our proposed method with respect to various methods.

**Table 1:** PSNR (dB) evaluation of different algorithms under two noise levels ($\sigma = 15$, $\sigma = 20$) on the Middlebury dataset.

Datasets	Art		Reindeer		Dolls		Book		Laundry		Moebius		Average	
Method	$\sigma = 15$	$\sigma = 20$	$\sigma = 15$	$\sigma = 20$	$\sigma = 15$	$\sigma = 20$	$\sigma = 15$	$\sigma = 20$	$\sigma = 15$	$\sigma = 20$	$\sigma = 15$	$\sigma = 20$	$\sigma = 15$	$\sigma = 20$
BF^1^	36.97	35.67	35.69	34.39	35.62	34.21	35.85	34.53	36.26	34.97	35.99	34.66	36.06	34.74
TV^22^	37.71	36.31	37.22	35.77	36.99	35.46	37.05	35.52	37.56	36.09	37.12	35.61	37.28	35.80
K-SVD^7^	38.13	36.77	37.66	36.29	37.37	35.97	37.55	36.05	37.97	36.57	37.33	35.93	37.67	36.26
BM3D^2^	38.96	37.35	38.25	36.73	38.09	36.56	38.12	36.57	38.61	36.99	38.04	36.43	38.35	36.77
CSF^14^	39.07	37.66	38.63	37.21	38.31	36.90	38.27	36.83	38.52	37.12	37.77	36.47	38.43	37.03
TGV^24^	39.23	37.73	38.67	37.16	38.61	37.12	38.56	37.05	38.92	37.40	38.33	36.82	38.72	37.21
WNNM^6^	39.35	37.85	**39.36**	37.81	38.91	37.31	38.82	37.33	39.01	37.50	38.44	36.87	38.98	37.45
TNRD^13^	**39.52**	37.91	39.26	37.74	38.89	37.44	38.87	37.27	39.09	37.55	**38.62**	37.05	39.04	37.50
MLP^12^	39.36	**37.95**	39.27	**37.93**	**38.97**	**37.51**	**39.09**	**37.79**	**39.11**	**37.71**	38.56	**37.26**	**39.06**	**37.69**
ESTGV	*39.57*	*38.06*	*39.44*	*38.02*	*39.29*	*37.97*	*39.33*	*38.01*	*39.39*	*37.81*	*39.07*	*37.59*	*39.35*	*37.91*

Italics indicates the best, bold indicates the second best, underscore indicates the third best.

**Table 2:** PSNR (dB) evaluation of different algorithms under two noise levels ($\sigma = 25$, $\sigma = 50$) on the Middlebury dataset.

Datasets	Art		Reindeer		Dolls		Book		Laundry		Moebius		Average	
Method	$\sigma = 25$	$\sigma = 50$	$\sigma = 25$	$\sigma = 50$	$\sigma = 25$	$\sigma = 50$	$\sigma = 25$	$\sigma = 50$	$\sigma = 25$	$\sigma = 50$	$\sigma = 25$	$\sigma = 50$	$\sigma = 25$	$\sigma = 50$
BF^1^	34.52	31.52	33.25	30.26	33.07	30.07	33.39	30.39	33.82	30.85	33.52	30.53	33.60	30.60
TV^22^	35.15	33.35	34.61	32.81	34.31	32.50	34.37	32.56	34.95	33.13	34.47	32.65	34.64	32.83
K-SVD^7^	35.61	33.80	35.13	33.32	34.81	33.01	34.89	33.08	35.41	33.62	34.77	32.96	35.10	33.30
BM3D^2^	36.18	34.39	35.56	33.77	35.39	33.62	35.4	33.63	35.82	34.03	35.26	33.44	35.60	33.81
CSF^14^	36.50	34.69	36.05	34.22	35.74	33.95	35.67	33.86	35.96	34.16	35.31	33.52	35.87	34.07
TGV^24^	36.57	34.75	35.97	34.18	35.96	34.16	35.89	34.07	36.26	34.44	35.67	33.85	36.05	34.24
WNNM^6^	36.68	34.87	**36.65**	34.85	36.13	34.33	36.16	34.35	36.32	34.52	35.69	33.87	36.27	34.47
TNRD^13^	36.71	34.92	36.52	34.75	36.26	34.44	36.05	34.27	**36.51**	**34.71**	35.87	34.16	36.30	34.54
MLP^12^	**36.75**	**34.95**	36.73	**34.92**	*36.31*	**34.52**	**36.59**	**34.82**	36.49	34.71	**36.06**	*34.33*	**36.49**	**34.71**
ESTGV	*36.86*	*35.07*	*36.82*	*35.02*	**36.30**	*34.67*	*36.81*	*35.03*	*36.61*	*34.82*	*36.39*	**34.31**	*36.71*	*34.82*

Italics indicates the best, bold indicates the second best, underscore indicates the third best.

As shown in Tables 1 and 2, the PSNR results of each denoising algorithm on different depth maps are different due to the internal structure variance of the six datasets, and the proposed ESTGV achieves the highest PSNR values on different depth maps in the vast majority of the cases, which shows significant improvement in comparison to other denoising methods. The second-best denoising algorithm i.e. MLP^12^, despite outperformed the proposed ESTGV slightly on Dolls dataset at $\sigma = 25$ and Moebius dataset at $\sigma = 50$, with only 0.01 dB and 0.02 dB, respectively, it underperformed the proposed ESTGV model on all datasets with two noise levels of $\sigma = 15$ and $\sigma = 20$. Our results verify that the proposed Edge-guided Second-order Total Generalized Variation (ESTGV) can provide more superior performance for depth map denoising. Our results also indicate that MLP^12^ is suitable for training a separate denoising model for each individual noise level. However, if the noise level of the test dataset deviates from the trained dataset, its performance would be compromised and it is impractical to train a separate MLP^12^ denoising model for each noise level. On average, the proposed ESTGV achieves 0.11 dB, 0.22 dB, 0.29 dB and 0.22 dB performance improvements over MLP^12^ at four individual noise levels ($\sigma = 20$, $\sigma = 25$ and $\sigma = 50$), respectively. Compared with the benchmark BF method^1^, the proposed ESTGV has improved 3.29 dB, 3.17 dB, 3.11 dB and 4.22 dB, respectively. In summary, our results show that the proposed ESTGV can provide robust performance at different noise levels in comparison to its counterparties.

**Figure 1:**
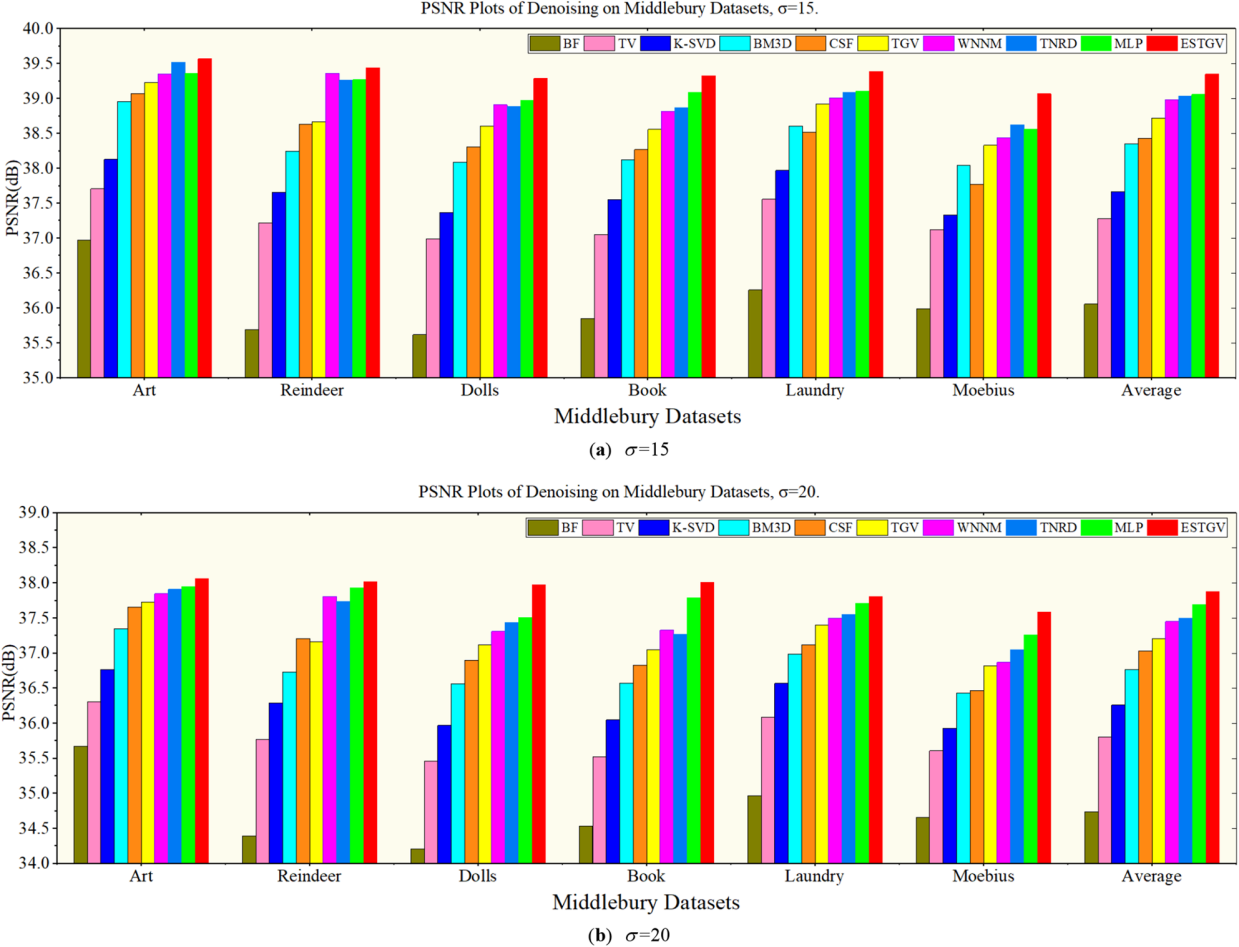
PSNR (dB) results of different denoising methods on Middlebury datasets ($\sigma = 15,\;\sigma = 20$).

**Figure 2:**
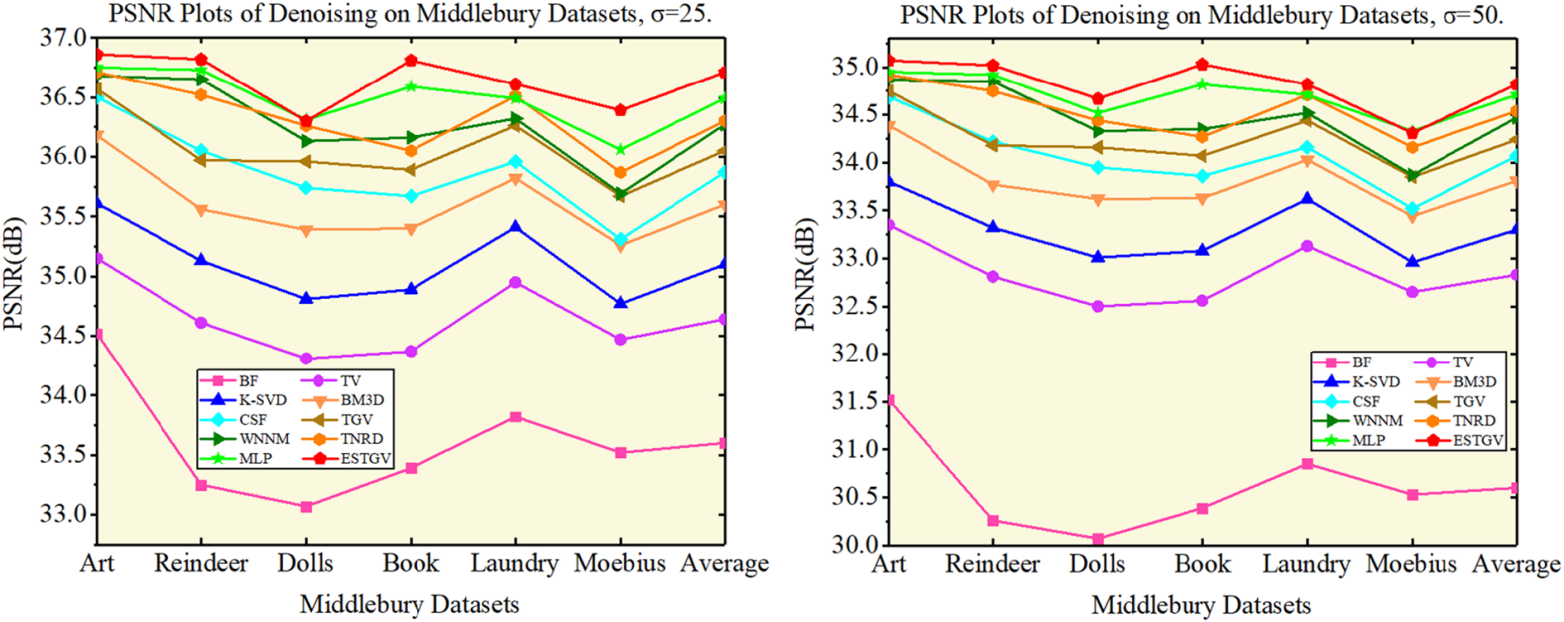
PSNR (dB) results of different denoising methods on Middlebury datasets ($\sigma = 15,\;\sigma = 20$).

Figures 1 and 2 show intuitively the results of removing four levels of Gaussian noise from the Middlebury dataset^32^ by 10 algorithms in the form of histogram and broken line graph, respectively.

### Qualitative results

**Figure 3:**
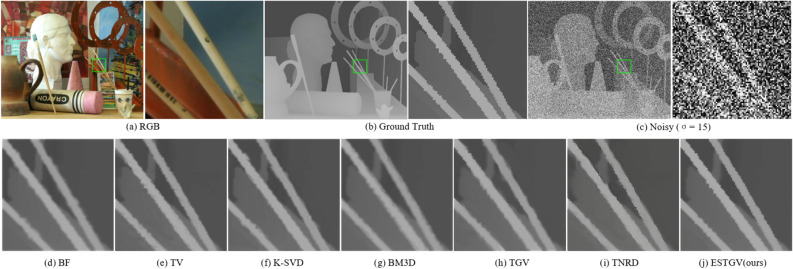
Denoising results on depth map *Art* by different methods (noise level $\sigma = 15$).

**Figure 4:**
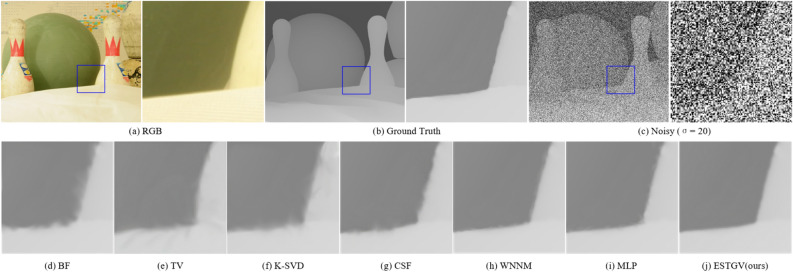
Denoising results on depth map *Bowling* by different methods (noise level $\sigma = 20$).

**Figure 5:**
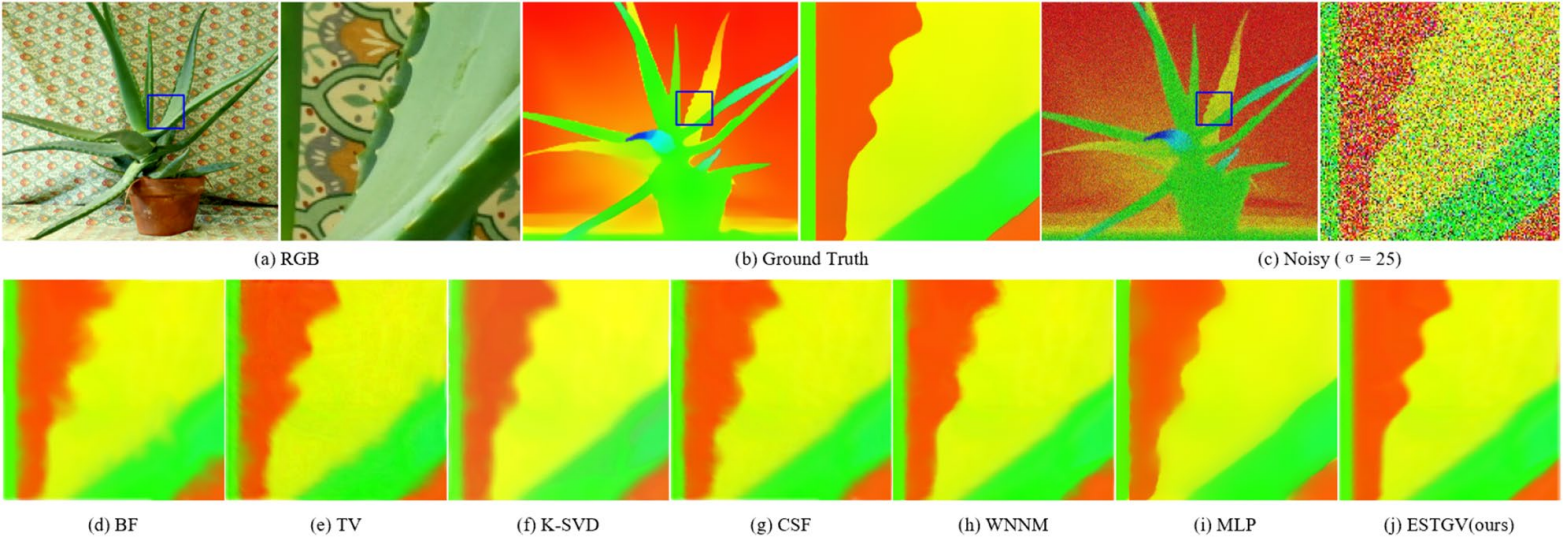
Denoising results on depth map *Aloe* by different methods (noise level $\sigma = 25$).

**Figure 6:**
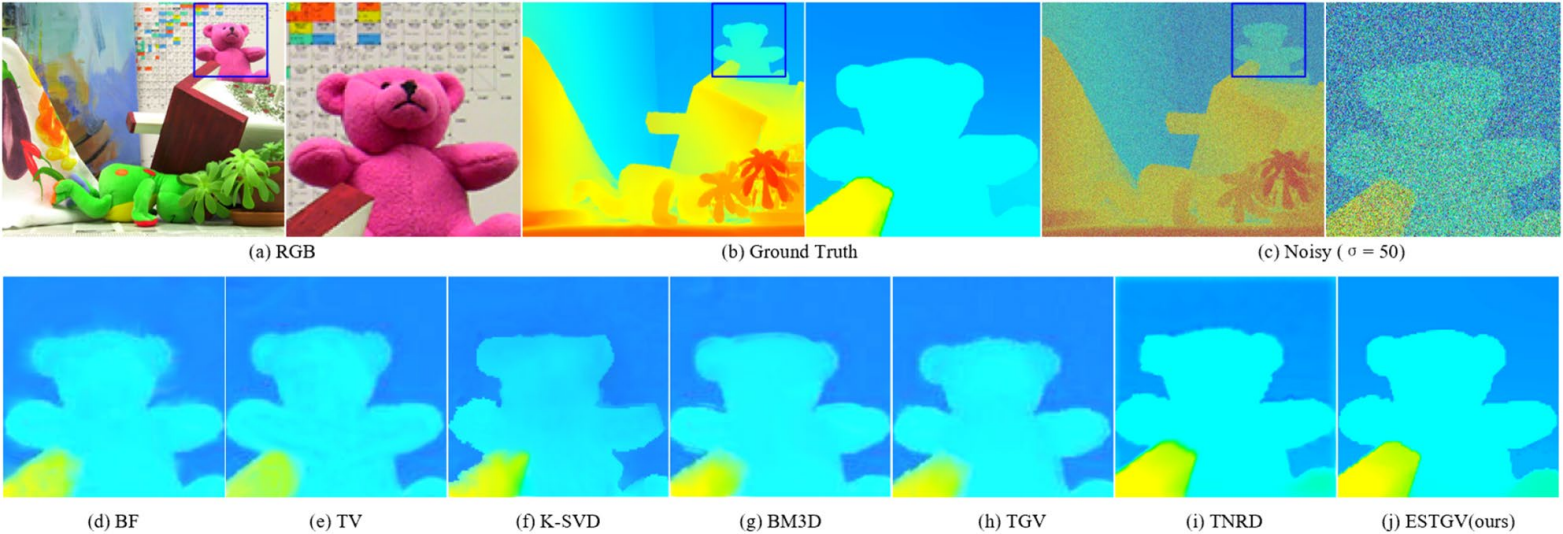
Denoising results on depth map *Teddy* by different methods (noise level $\sigma = 50$).

We evaluate the proposed ESTGV method visually in Figs. 3, 4, 5, 6, which show the visual comparison of different denoising methods on *Art, Bowling, Aloe* and *Teddy* from Middlebury dataset, respectively. We have the following observations. MLP^12^ over-smooths the textures more on the edges of depth map. TNRD^13^ can reconstruct sharp edges and fine details, but it is easy to generate artifacts in smooth areas. Methods BM3D^2^ and CSF also generate blurred boundaries and block artifacts. Although WNNM^6^ has a proper balance between noise removal and edge preservation, it can still cause the depth details to be too smooth and produce ringing phenomena. K-SVD^7^ can preserve clear edges and rich details, but it also generates structural distortion phenomena such as jags and burrs in the edge area. TV^22^ produces obvious “staircasing artifacts”. Compared with TV^22^, TGV^24^ can avoid “staircasing artifacts” and appear smoother and more natural in visual effect, but it cannot preserve the edges too well because of its over-smoothness reduces the sharpness of the depth map causing some blocky effect in the smooth area. The result of BF^1^ is the blurriest with texture artifacts existing in various areas of the depth map. It is obvious that the proposed ESTGV generates much less artifacts and preserves the edge details much better than other denoising algorithms. In summary, the proposed ESTGV algorithm shows more robustness to noise strength, producing much more pleasant visual outputs.

## Conclusions

Aiming to extend the research in improving generalized variational models’ denoising capacity on depth maps, we have presented an edge-guided second-order total generalized variation model for Gaussian noise removal (ESTGV) in this paper. ESTGV fuses an edge indicator function into the regularization term of the second-order total generalized variational model to guide the diffusion of gradients. It can adaptively use the first or second derivative to update the intensity of the diffusion tensor, and therefore effectively denoise and preserve edges under different intensity noise levels. Our quantitative and qualitative experimental results demonstrated that the proposed ESTGV method shows more robustness on noise strength in comparison to very recent state-of-the art denoising algorithms. It can not only lead to visible PSNR improvements over state-of-the-art methods such as MLP, WNNM and TNRD, but also preserve the depth structures much better and generate much less texture artifacts.
